# Peroneal Arteriovenous Fistula and Pseudoaneurysm: An Unusual Presentation

**DOI:** 10.1155/2014/506067

**Published:** 2014-09-30

**Authors:** Kevin C. Ching, Kevin M. McCluskey, Abhay Srinivasan

**Affiliations:** ^1^Department of Radiology, University of Pittsburgh, 3950 Presby South Tower, 200 Lothrop Street, Pittsburgh, PA 15213, USA; ^2^Department of Radiology, Children's Hospital of Pittsburgh, Pittsburgh, PA 15213, USA

## Abstract

Peroneal artery arteriovenous fistulas and pseudoaneurysms are extremely rare with the majority of reported cases due to penetrating, orthopedic, or iatrogenic trauma. Failure to diagnose this unusual vascular pathology may lead to massive hemorrhage or limb threatening ischemia. We report an interesting case of a 14-year-old male who presented with acute musculoskeletal pain of his lower extremity. Initial radiographs were negative. Further imaging workup revealed a peroneal arteriovenous fistula with a large pseudoaneurysm. After initial endovascular intervention was unsuccessful, the vessels were surgically ligated in the operating room. Pathology revealed papillary endothelial hyperplasia consistent with an aneurysm and later genetic testing was consistent with Ehlers-Danlos syndrome Type IV. This case illustrates an unusual cause of acute atraumatic musculoskeletal pain and uncommon presentation of Ehlers-Danlos syndrome.

## 1. Introduction

Arteriovenous fistulas often result from iatrogenic trauma while gaining percutaneous vascular access. Turbulent blood flow and endothelial damage may eventually lead to pseudoaneurysm formation with resulting mass effect and risk of massive hemorrhage. While several case reports have documented peroneal pseudoaneurysms resulting from iatrogenic and orthopedic trauma, there are no known reports of both an arteriovenous fistula and pseudoaneurysm presenting in a patient with an undiagnosed connective tissue disorder. Utilizing multiple imaging modalities, this case nicely documents the unique vascular findings in this interesting case.

## 2. Case Report

**Figure 1:**
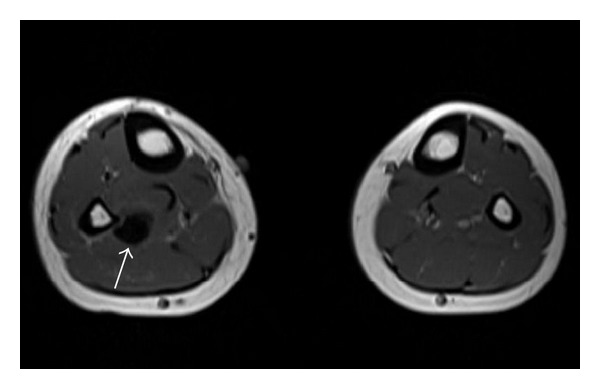
Axial T1 MRI of the lower extremities demonstrates a 1.6 × 1.2 × 1.5 cm signal void* arrow* adjacent to the right peroneal artery and vein.

**Figure 2:**
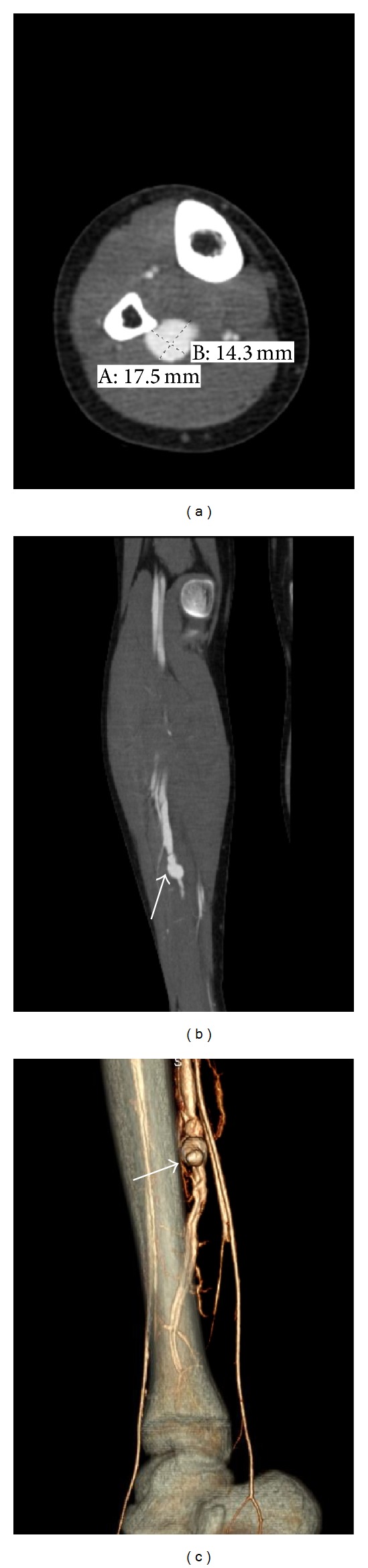
CT angiogram of the right lower extremity. (a) Axial, (b) coronal, and (c) volume rendered images show a 1.8 × 1.4 cm contrast filled pseudoaneurysm* arrow* with a direct communication between the peroneal artery and peroneal vein. Early venous filling indicated an arteriovenous fistula.

**Figure 3:**
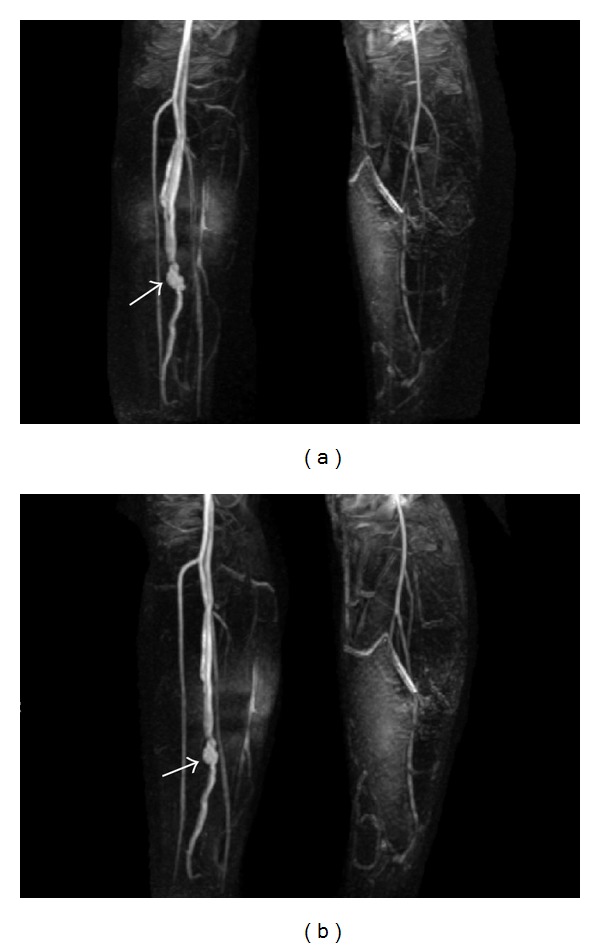
MR angiogram with contrast. (a) AP and (b) oblique images demonstrate the peroneal artery pseudoaneurysm and early filling* arrows* of the venous system.

A healthy 14-year-old male presented with acute onset lower extremity calf pain and swelling that began while playing soccer. He denied trauma to the limb and on physical exam was neurovascularly intact. An MRI to evaluate possible ligamentous injury revealed a large abnormal flow void adjacent to the peroneal artery and vein thought to represent a pseudoaneurysm or arteriovenous malformation (Figure 1). CT angiogram of the lower leg showed a direct communication between the peroneal artery and vein with early filling of the peroneal and popliteal veins indicating an arteriovenous fistula (Figure 2). There was also a focal dilatation at the communication of the vessels compatible with a pseudoaneurysm. These findings were redemonstrated on contrast enhanced MR angiogram (Figure 3).

**Figure 4:**
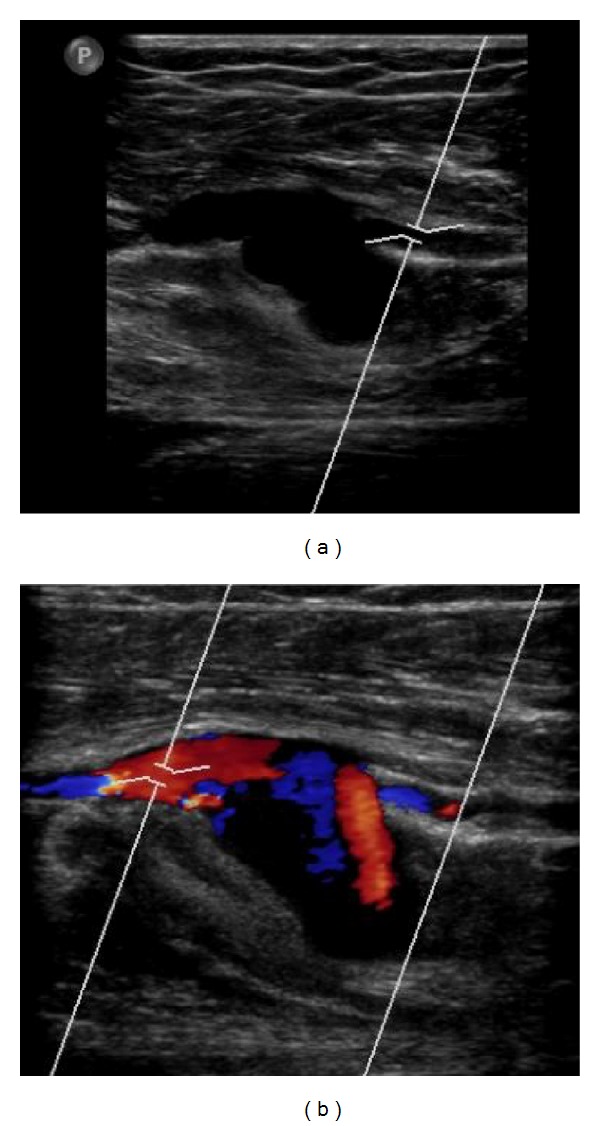
Ultrasound (a) Gray-scale and (b) Color Doppler images show the peroneal artery and veins directly entering the pseudoaneurysm sac. Swirling of blood flow on Color Doppler results in the characteristic “Yin-Yang” appearance.

**Figure 5:**
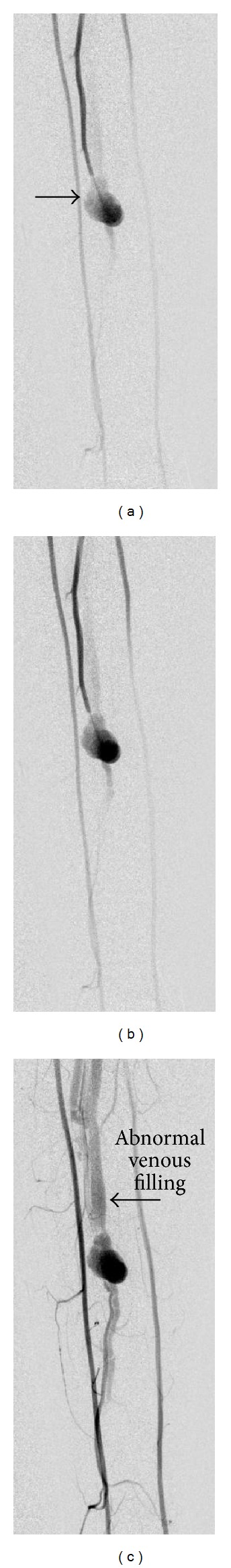
Digital subtraction arteriogram (a–c) shows filling of the wide-necked peroneal pseudoaneurysm (b) with arteriovenous fistula to the peroneal vein (c).

Ultrasound showed a 2.0 × 1.5 cm pseudoaneurysm with swirling of blood flow in the pseudoaneurysm sac giving the characteristic “Yin-Yang” appearance on Color Doppler (Figure 4). Both peroneal artery and vein were shown to directly communicate with the aneurysm sac. Rapid arterialized blood flow was present in the peroneal vein and rapid low resistance flow was present in the peroneal artery. Digital subtraction angiography confirmed the large wide-necked pseudoaneurysm originating from the peroneal artery with an encompassing arteriovenous fistula to the peroneal vein (Figure 5).

Percutaneous intervention was attempted; however, a wire could not be successfully placed distally in the peroneal artery to traverse the pseudoaneurysm neck. The patient subsequently underwent open ligation of the pseudoaneurysm and arteriovenous fistula. Postoperatively his symptoms resolved and genetic analysis revealed a collagen mutation compatible with Vascular Ehlers-Danlos syndrome Type IV.

## 3. Discussion

Arteriovenous fistulas (AVF) and pseudoaneurysms (PSA) of the peroneal artery are uncommonly reported in the literature and are extremely rare presenting together. Most pseudoaneurysms result from orthopedic, penetrating, or iatrogenic trauma with the latter often seen after Fogarty balloon embolectomy or percutaneous vascular access [1, 2]. Congenital AVF can be seen in the peripheral vasculature and are usually completely asymptomatic; however, significant shunting may occur [3].

While the pseudoaneurysm and arteriovenous fistula in this patient were initially thought to be traumatic in origin, genetic testing later revealed a mutation in the COL3A1 gene of collagen 3 compatible with a diagnosis of the Vascular (Type 4) Ehlers-Danlos syndrome. Of the 6 major types of Ehlers-Danlos syndrome, Type 4 is one of the most serious types and is most often associated with major vascular complications [4]. When evaluating a patient for Vascular Ehlers-Danlos, Loeys-Dietz syndrome Type 2 should also be considered. Loeys-Dietz syndrome (LDS) is a recently discovered aortic tortuosity syndrome due to mutations in transforming growth factor beta receptors 1 and 2 (TGFBR1/2) [5]. While vascular pathology in LDS is similar to Vascular Ehlers-Danlos syndrome, LDS Type 2 presents with characteristic craniofacial malformations such as cleft palate, hypertelorism, and craniosynostosis.

Peroneal artery pseudoaneurysms have been successfully treated by coil embolization of the pseudoaneurysm sac or, alternatively, embolization of the peroneal artery proximally and distally to the pseudoaneurysm neck [6, 7]. While ischemia of the lower extremity is a potential risk when embolizing the peroneal artery, it is unlikely to occur with sufficient collateral flow from nondiseased anterior and posterior tibial arteries [8]. When sacrificing the vessel is not an option, peroneal arteriovenous fistulas may be percutaneously treated with a stent graft to exclude the fistula [3]. There is at least one successful report in the literature of a 3 mm coronary stent graft deployed in the peroneal artery to exclude the fistula and maintain distal vessel patency. In summary, peroneal arteriovenous fistulas with pseudoaneurysm are extremely uncommon and when they are encountered without an identifiable traumatic etiology, an evaluation for an underlying connective tissue disorder should be performed.
